# Sociodemographic factors and treatment effects on quality of life in locally advanced breast cancer: a cross-sectional study

**DOI:** 10.3332/ecancer.2025.1965

**Published:** 2025-08-15

**Authors:** Sasongko Hadi Priyono, Winardi Budiwinata, Budianto Tedjowitono, Effendy, Muhamad Daffa Ibnurasy Pratama

**Affiliations:** 1 Department of Surgical Oncology, Ulin General Hospital, Faculty of Medicine, Universitas Lambung Mangkurat, Banjarmasin 70123, Indonesia; 2 Department of Surgery, Ulin General Hospital, Faculty of Medicine, Univeritas Lambung Mangkurat, Banjarmasin 70123, Indonesia; 3 Faculty of Medicine, Universitas Lambung Mangkurat, Banjarmasin 70123, Indonesia; a https://orcid.org/0009-0006-0212-2513; b https://orcid.org/0009-0005-8754-0302; c https://orcid.org/0009-0002-9763-8862; d https://orcid.org/0000-0003-3094-4646; e https://orcid.org/0009-0001-5277-3369

**Keywords:** breast neoplasmsm, quality of life, neoplasm

## Abstract

**Purpose:**

This study aimed to identify key aspects of health-related quality of life in women with locally advanced breast cancer (LABC) and analyse their links to factors and treatment modalities.

**Method:**

A cross-sectional study was conducted from August to October 2023 in Ulin Regional Public Hospital, Banjarmasin, Indonesia, involving LABC women whose quality of life (QoL) was assessed using Quality-of-Life Questionnaire Breast Cancer 23. Data were analysed using ANOVA, independent t-tests for parametric data, Kruskal-Wallis and Mann-Whitney tests for non-parametric data and significant variables (*p* < 0.05) included in a final regression model for identifying predictors.

**Results:**

Of 100 participants (mean age 50 years), most had low education levels (41%), were unemployed (74%) and had stage IIIB cancer. Body image score was the highest, while systemic therapy side effect was the lowest. Better sexual enjoyment was reported in post-menopausal women (*p* = 0.043), those with higher education (*p* = 0.036) and married individuals (*p* = 0.021). Higher economic status was associated with better sexual enjoyment (*p* = 0.008) and fewer breast symptoms (*p* = 0.011); however, economic status was negatively associated with employment status (*p* = 0.043). Worsening arm symptoms were associated with prolonged illness (*p* = 0.022). Surgical intervention was associated with higher body image (*p* = 0.010) and lower systemic side effects (*p* = 0.023). Traditional medicine was associated with lower arm symptoms (*p* = 0.026). Economic/occupational status explained 10.5% of sexual functioning scores.

**Conclusion:**

Poor QoL in LABC patients overall was associated with low sociodemographic conditions, late presentation and chemotherapy-related side effects.

## Introduction

Breast cancer is the most common cancer in women globally, with 2.3 million cases diagnosed in 2022, causing 665,000 deaths globally, as reported by the WHO. Up to 2022, half of the breast cancer cases were diagnosed in Asia, where it ranked second after lung cancer but was the most common cancer among women [1, 2]. In Indonesia, breast cancer accounted for 30.8% of all female cancer cases and 20.4% of cancer-related deaths in 2020, with projected increases in incidence and mortality rates regarding late diagnosis and limited access to treatment [3].

In low- and middle-income countries (LMICs), breast cancer often presents at a locally advanced stage (LABC) and research on quality of life in women with LABC in LMICs, particularly in Indonesia, where breast cancer is frequently diagnosed at later stages with poorer prognosis-remains limited [4–7]. LABC, classified into stages IIIA, IIIB and IIIC according to the TNM system (T = tumour, N = regional lymph nodes spread and M = metastasis), is characterised by tumours larger than 5 cm, skin or pectoral muscle involvement, lymph node involvement or inflammatory breast cancer, without distant metastases [8].

Despite being classified as ‘locally advanced,’ LABC often signifies early rapid metastasis. Breast cancer development is multifactorial, significantly impacting patient quality of life (QoL). [9] Health-related quality of life (HR-QoL) assessments are crucial for evaluating disease severity, progression and treatment impact. QoL may be influenced by clinical manifestations and psychological effects, including depression [10]. Advanced malignancies often correlate with impaired functioning and cancer-related life disruption, requiring rehabilitation, yet in developing nations like Indonesia, these services are often inaccessible or insufficient due to provider awareness [11, 12].

At the regional level, Asian patients have a lower HR-QoL than their Western counterparts and Asian patients tend to avoid private topics and dislike discussing them in public, which presents a challenge for healthcare professionals [13]. Present studies regarding QoL on breast cancer in Indonesia are mostly done in the central administrative area of Java, which culminated in a lack of data from patients from outside of Java and are mostly done on unspecified breast cancer presentations [14]. Social structure, culture and beliefs amongst the populace differed across Asian countries, notably Indonesia, with its diverse population, which often contrasts with the more sophisticated Java, and to add, scarce data obtained from Kalimantan may provide further studies regarding cancer in Indonesia [15].

This study aims to evaluate how different factors, including sociodemographic characteristics and disease progression, are associated with changes in the QoL of women with LABC and analyse the association between QoL and these parameters.

## Materials and Methods

### Study design and population characteristics

This is an analytical cross-sectional study involving women aged 30 years and older admitted and diagnosed with LABC across all stages (IIIA, IIIB and IIIC). The study was performed at the Regional Public Hospital Ulin in Banjarmasin, Indonesia, a tertiary hospital that provided services for two provinces at once. Despite this, there is still no multidisciplinary team approach from the beginning of patient care towards cancer case management; cases are often handled only by a single department. Our center also has both rehabilitation and psychological services available; however, it should be noted that these services are not integrated regularly into patient management. As of our research, no specific palliative care unit was available exclusively for both early and end-stage breast cancer patients.

The calculated sample size for this study was 90, obtained from the Lemeshow formula. A post-hoc power analysis was conducted to statistically verify the use of a sample size of 100 participants. This result was validated under the conditions of having an alpha level of 0.05, assuming adherence to statistically significant thresholds of 0.80 and identification of a medium-sized effect (f = 0.25). This analysis confirmed that the achieved power was adequate to identify large, medium-sized effects in group comparisons of quality-of-life parameters using the Quality-of-Life Questionnaire Breast Cancer 23 (QLQ-BR23) instrument.

By examining the sample population in the regional health survey data, it was found that Southeast Asia experiences significant late-stage presentation burdens, as evidenced in the 2020 study conducted by the ASEAN Cost in Oncology project, which found that socioeconomic disparities, low screening uptake and late health-seeking behaviours were among the most significant contributors to the advanced stage presentation in Southeast Asia [16].

Our findings provide evidence that our sample population is a true representation of the wider regional population, both in age and socioeconomic status and educational disparities.

The study population consisted of women with breast cancer admitted to the surgical oncology service between August and October 2023, totaling 100 patients. It was suggested that younger women report significantly different perspectives on body image and distinct psychological conditions; hence, to avoid bias, we excluded women younger than 30 years old [17]. The inclusion criteria, therefore, were women aged 30 years or older, diagnosed with locally advanced breast cancer, i.e., in clinical stages III (A, B and C)–while patients with distant metastases were excluded. Our study population primarily consisted of patients from low-income households, both urban and rural, as our center serves as the only end-referral hospital in the region.

Anamnesis, physical examination, imaging studies and histopathological examinations were done to diagnose them as eligible for the study.

Patients were recruited during their first appointment at the surgical oncology service. Those who agreed to participate in the study signed an informed consent form and were interviewed using the QLQ-BR23 questionnaire by designated researchers. The QLQ-C30 was excluded due to the high number of English terms in the CLC-Q30, which were difficult for the Indonesian population, particularly those with limited education, to understand. These terms often did not align with the Bahasa Indonesia translation or local language equivalents. As a result, fatigue and global health status were not assessed in this study. We achieved an 80% response rate, with refusals primarily due to participants’ difficulty understanding the questions or lack of time for the interview.

The administered QLQ-BR23, used as a QoL response parameter to the disease, is a supplementary questionnaire for breast cancer patients using the Bahasa Indonesia version. This questionnaire has 23 questions, divided into two dimensions: a functional scale (body image, sexual functioning, sexual enjoyment and future perspective) and a symptom scale (systemic therapy side effects, breast symptoms, arm symptoms and upset by hair loss). The Bahasa Indonesia version of the questionnaire has been proven to be valid and reliable for usage [18].

Clinical data, including time from initial clinical presentation until the last period of treatment, is also collected along with the history of medication that the patient has taken: hormonal therapy, chemotherapy, surgical procedures and other unconventional medicines not prescribed by the clinician, limited to the usage of traditional and herbal medicines. Our data regarding these were collected during our primary verbal interview and several data were confirmed with medical records. Sociodemographic data was also collected during the primary verbal interview obtained from patients and their families to provide clarity on several key items such as monthly incomes, decline in savings and livelihood difficulties. For this, we provided separate specified questionnaires in addition to the BR-23 questionnaire, with question items such as: (1) What is your average family monthly income?

(2) Does breast cancer impact your savings?

(3) Has your capability to purchase daily goods been impaired since contracting cancer?

### Data analysis

The data were coded and analysed with the Statistical Package for the Social Sciences (SPSS) using IBM SPSS Version 26. EORTC BR-23 was scored according to the rules established in the EORTC manual.

The analysis of the results targeted points by which QoL is/are affected. This evaluation is then compared between three stages of LABC (IIIA, IIIB and IIIC); hypothesis tests were proposed for more than two populations, in which values of *p* < 0.05 (established significance level) were considered as significant results.

The Kolmogorov-Smirnov test was performed to verify the assumption of data normality, in addition to graphical analysis using histograms to analyse questionnaire responses.

The scores obtained from both functional scale and symptom scale parameters were the dependent variables in the study, while the basic cancer staging and several specified parameter groups, ranging from age, menopausal status, marital status, therapeutic surgery and sociodemographic status, were the independent variables. The ANOVA and independent *t*-test parametric tests were used for the categorical variables and the Kruskal-Wallis and Mann-Whitney tests were used for non-parametric tests.

Additional analytical testing was done to measure each predictor parameter's significant correlation and to calculate the coefficient of determination using a linear regression test. The dependent variables were the symptom scale and functional scale. While cancer staging, age, menopausal status, marital status, educational status, occupational status, economic status and in clinical parameter group, were chosen as independent variables and were labeled into each of their own group variables and considered as the models’ predictors. The value of R squared was calculated, and *p* ≤ 0.05 was taken as significant where the comparison was conducted.

### Ethical considerations

An exploratory interview was done, including but not limited to informed consents, which included that the patient’s data from the interview may be utilised for research purposes. The data obtained from interviews using the QLQ-BR23 questionnaire has been fully anonymised for each patient. Patients were recruited at their first appointment at the Oncology Surgery service. Those who agreed to participate in the study were asked to sign the informed consent form and were interviewed by appointed researchers.

The joint ethics committee from the Faculty of Medicine of Universitas Lambung Mangkurat/Ulin Regional Public Hospital has acknowledged and agreed on this use of primary data.

## Results

### Sociodemographic and epidemiological characteristics

In total, 100 women were interviewed for their responses. The mean age of participants was 50 years (SD ± 9.98 years).

The participants exhibited diverse educational backgrounds, with categorisation aligned with the Indonesian formal education system. A significant proportion of the female participants (41%) attained the lowest strata of education, completing 0–6 years of schooling (Table 1).

Analysis of sociodemographic elements, including occupational status, revealed a high unemployment rate (74%). The financial element, using 3 parameters, showed that 70% reported income below minimum wage, two-thirds (67% versus 33%) with reduced savings and near-equal reports on daily livelihood difficulties (49% versus 51%). These indicate that most participants experience financial hardship (Table 1).

### Clinical data

Clinical data revealed that nearly all the patients had stage IIIB disease (89%) and approximately half were diagnosed between 1 and 5 years. Regarding treatment modalities, chemotherapy was administered for most subjects. Modified-radical mastectomy was performed on 44% of the patients. Notably, 21% of the patients had resorted to non-clinician-recommended treatments during their disease course (Table 2).

### Assessment of quality of life using QLQ-BR-23 questionnaires

**Figure 1.**
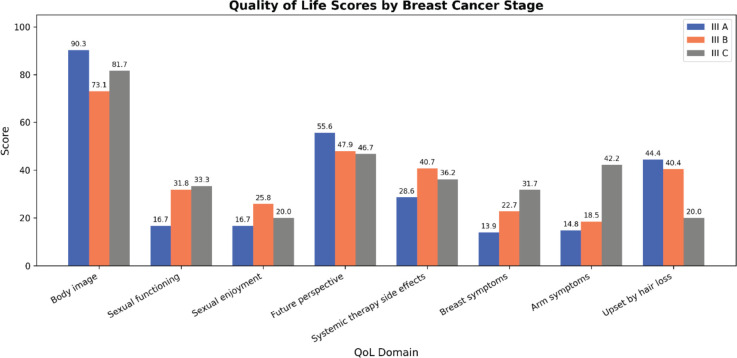
Results of both functional scales and symptom scales between clinical stages of locally advanced breast cancer.

Overall, QLQ-BR-23 variables are depicted in Table 3. Most participants consistently reported higher scores for body image, as evidenced by a high mean score and a low standard deviation. A high standard deviation suggests that both sexual enjoyment and functioning afflicting these women differ greatly from one another. Future perspective declined in the future perspective group with advancing clinical stage (Figure 1).

We also classified our patients into two groups: Scores of ≤ 33 in functional scales indicated problematic function, while scores ≥ 66 suggested better QoL; this scoring was reversed in symptom scale assessment based on grouping method done by Imran *et al* [19] on their earlier study on breast cancer patient population as to better identify patients with more QoL issues with score-based approach. Based on that, patients exhibited better QoL during the treatment. [18] However, sexual-related QoL was lower, with a high standard deviation indicating significant inter-patient variability.

### Comparative analysis of QLQ-BR-23 quality of life scores between parameters

QLQ-BR-23 QoL scores were compared across demographic and clinical groups. Functional scale depicted similar trends, showing higher body image and future perspective in stage IIIA compared to other stages, though declines with advancing stages were not statistically significant. Likewise, arm and breast symptoms increased with advancing clinical stage, though not significantly.

Age did not considerably affect QoL scoring; however, the ≥50 years subgroup experienced lower functional scores and higher symptom scores. Illness duration correlated with decreased symptom severity, relative to those without symptoms, over time, except for arm symptoms, which worsened significantly after 5 years (*p* = 0.026). Patients treated with surgical interventions demonstrated better QoL: higher functional scores, particularly for body image (*p* = 0.010) and reduced incidence of systemic therapy side effects (*p* = 0.023) and breast symptoms (*p* = 0.035).

Demographic, socioeconomic and net health status factors significantly influenced sexual enjoyment. Relationship status has a significant effect, with married participants reporting higher sexual satisfaction than unmarried ones (*p* = 0.000). Menopausal status correlated with increased sexual enjoyment (*p* = 0.043), suggesting hormonal or life stage influences. Sexual enjoyment increased with education duration (highest scores = highest education; *p* = 0.036) [27] with a slight decline at the highest education level. Higher income (above minimum wage) was associated with greater sexual enjoyment (*p* = 0.008). Conversely, employment status negatively impacted sexual enjoyment (*p* = 0.043) (Table 4).

Multivariate analysis showed that occupational and economic status explained 10.5% of the variance in sexual functioning (adjusted R-squared = 0.105). Marital status was the most significant indicator of sexual enjoyment, explaining 18.5% of the variation (*p* = 0.004) (Tables 5 and 6).

Use of alternative therapies (traditional or herbal medicines) showed no correlation with functional scale scores but was associated with lower arm symptoms (*p* = 0.026) and a better future perspective.

Quality of life is determined by a wide variety of factors, such as surgical intervention and sociodemographic factors. These findings will aid in the development of targeted interventions aimed at enhancing individual quality of life, sexual well-being and symptom management.

## Discussion

Our study found that body image was the least disturbing on the functional scale, while sexual functioning and enjoyment had the lowest scores and highest variability. We identified several HR-QoL aspects that were associated with sexual aspects of QoL in our patients. Early menopause and being not married were linked to both reduced sexual functioning and enjoyment, in addition to worse breast symptoms, respectively. Higher education level and economic status were associated with better sexual enjoyment and breast symptoms, while higher symptoms were associated with unmarried patients. Modified radical mastectomy (MRM) patients were also associated with higher body image, in addition to lower breast symptoms and chemotherapy side effect score. Higher arm symptoms were associated with longer illness duration, and unconventional medicine usage was associated with lower arm symptom scores.

Our findings on improved body image and varied sexual aspect scores align with a Saudi Arabian study that also found higher scores in body image [19]. Similarly, a Chinese study showed high sexual functioning and enjoyment scores with a high standard of deviation, suggesting hesitation in discussing sexual topics [20]. Bobrie *et al* [21] emphasised that sexual issues are often overlooked in breast cancer care, exacerbated by inadequate communications. In Indonesia, hesitance by professionals was also found in addressing sexual issues [22]. Inadequate professional awareness can exacerbate proof sexual QoL, potentially leading to a cluster of psychological issue, including sexual dysfunction and depression [23]. Other studies also found that psychological issues persist in long-term breast cancer survivors, even with good QoL, underscoring the importance of addressing emotional needs in palliative care settings. Thus, interventions aiming to address psychological issues and sexual problems that are focused on the active participation and counselling by psychologist, sexual health counsellors and psychiatrists, may be beneficial towards improving QoL in breast cancer patients [24, 25]. In this aspect, there was still no integrated multidisciplinary approach towards psychological supports in Indonesia as of during the time of our study.

Several HR-QoL aspects correlated with sexual aspects of QoL in our patients. Our findings on menopausal status align with Park and Yoon [26], who found that early menopause triggered by acute ovarian failure due to chemotherapy adversely affected sexual function and increased depression symptoms. Marschner *et al* [27] also reported that premenopausal women undergoing chemotherapy experienced more severe symptoms, including body image and emotional and social functioning. This again underscores the importance of physician and psychologist follow-up on post-chemotherapy sexual functioning in tackling other QoL-related problems. An Indonesian study found no significant association between menopausal status and sexual parameters but concurred that sexual issues remain inadequately addressed in Indonesia; however, the nature of cross-sectional model of our study should also be considered as to interpret this result despite various parameters associated [28]. A Malaysian study also suggested that many challenges to sexual wellbeing remained as an issue for cancer survivors living in settings with limited supportive care, citing that sexual issues are often overlooked in the planning of supportive care in LMIC settings [29]. These findings highlighted the need for professional counseling and the adoption of couple-focused sex therapy interventions, as they have been shown to be beneficial in fostering communication between couples and improving sexual relationships in both cancer patients and their partners [30, 31]. A couple-based approach is important in LMIC countries, mainly Asian countries, as marital relationships are often considered the primary framework for sexual activity and emotional intimacy in traditional societies. Chang *et al* [32] also suggested that emotional support from spouses enhanced QoL for breast cancer patients in China. However, other studies also indicated the need for more research to explore socio-economic issues like spousal dynamics, family economy and overall well-being related to a couple's stability [9, 33].

Our patients with higher education were associated with better sexual QoL, consistent with Telli [34], who associated this with better perception and an open perspective toward sexual practice. Some Indonesian studies done in central administration provinces did not find a significant impact of education on sexual enjoyment, possibly due to differences in patients’ demographics and regional educational distribution. These findings highlighted that breast cancer patients’ characteristics can also have further variations based on demographic status in each of a country’s regions, especially in LMICs, where both economic and social disparities are commonly found in contrast between central and peripheral regions, requiring a tailored approach to addressing multi-factorial QoL problems in local breast cancer patients [30, 35].

Our findings regarding chemotherapy aligned with studies done by Nguyen *et al* [36] and Cardoso *et al* [37], which noted that chemotherapy patients reported significant side effects, often worsened with disease progression. A Nigerian study linked worsening systemic therapy side effects to non-compliance with chemotherapy, often due to chemotherapy-induced anemia, neutropenia, lymphopenia and thrombocytopenia. [38] Prophylactic measures thus may be recommended to be integrated, but issues such as administration practices and high costs further complicate these treatment options in low-to-middle-income settings.

A review of studies involving breast cancer patients in low-to-middle-income Asian settings found that lower income significantly correlated with poorer overall QoL, consistent with our findings [39]. This contradicts Yusoff *et al* [40], who reported higher discomfort in higher socioeconomic groups. However, their study lacked specification of patient clinical stages, which may affect symptom presentation. A longitudinal study further addressed that low socioeconomic status significantly impaired follow-up care, HR-QoL and increased psychological distress in Southeast Asia populations [16]. Additionally, findings by Clegg-Lamptey *et al* [41] and Bichoo *et al* [42], further emphasised that fungating breast cancer, which often arises in developing countries, requires more complex treatment, raising costs and highlighted the need of coordinated multidisciplinary effort in addition to government initiative to alleviate economic burden and integration of social and occupational support groups tailored to LMICs breast cancer care.

MRM was associated with improved QoL in our study, potentially due to lower patient expectations in advanced disease stages and inherent expectations regarding MRM results compared to other types of surgeries [43, 44]. While MRM with neoadjuvant chemotherapy (NAC) improved certain QoL aspects, NAC alone was associated with diminished QoL due to increased toxicity, as observed in our patients [45, 46]. Thus, regular QoL assessments during chemotherapy are essential for monitoring and managing side effects, but it should also be noted that being a cross-sectional study, we cannot ascertain on the certainty that better QoL is always associated with combined therapy.

Arm symptoms showed mixed results: they decreased after 5 years of illness, contrasting with Norman *et al* [47], who reported worsening symptoms with prolonged duration. This discrepancy may stem from our patients’ arm symptoms being linked to reduced movement and subsequent inflexibility, which tends to improve with time [48]. Thus, additional tools may enhance arm symptom assessment in breast cancer studies. Traditional medicine use correlated with reduced arm symptoms in our study. Some studies suggested that this could be a perceived effect rather than an actual therapeutic benefit, acting as a coping mechanism. Others emphasized potential therapeutic effects, but further research is needed to validate the effectiveness of traditional medicines for LABC patients [49, 50]. Our patients utilised honey and *Phaleria macrocarpa* mixtures with several other unidentified substances for wound dressing for breast and flaky skin on their breast and arm. A randomised study done by Lund-Nielsen *et al* [51, 52] found honey-coated bandages effective for wound control; however, while these dressings reduced wound size, antimicrobial properties were not directly linked to improvement. The usage of *P. macrocarpa* was not directed by our physician; an *in-silico* study by Christina *et al* [53] suggested that bioactive compounds from *P. macrocarpa* leaf extract might potentially modulate signaling pathways related to cancer apoptosis and cell growth; however, they also noted that further research still should be performed to validate its efficacy for target cancer development. These findings implied that while usage of traditional medicines is often discouraged, mixed results implied that further research is needed to offer clarity, since evidence remains limited and caution is advised pending further research on its usage, since we cannot ascertain the causality of this association in our cross-sectional study.

Linear regression showed that marital status, occupation and economic status significantly predicted sexual functioning, with marital status being a strong predictor of sexual enjoyment. Our regression model findings align with Getu *et al* [54], where active marriage status predicted better sexual experience. Additionally, predictors for sexual functioning, such as menopausal status and sociodemographic factors, align with Smedsland *et al* [55] and another study from Saudi Arabia, where lower QoL was linked to worse sociodemographic conditions, where economic factors and occupational status also impacted sexual functioning. Patients with higher incomes and job stability experienced better sexual QoL [56]. These findings highlight the importance of socioeconomic stability in coping with breast cancer and sexual management should not be overlooked in breast cancer patients’ management and must be integrated to better improve patients’ overall well-being.

## Strength and Limitation

This is the first study in Indonesia to analyse multiple sociodemographic and disease parameters in relation to QoL, focusing on LABC in an LMIC context. However, limitations include the cross-sectional design and lack of a control group, which may affect the analysis. Furthermore, the usage of multiple comparisons is still a concern for further analysis adjustments. The exclusion of additional questionnaire items in our study is due to English terms that do not conform to terms contained in the Bahasa Indonesia further translation to local languages; thus, additional authorised questionnaire remained as a challenge, and thus, a questionnaire tailored to local languages can be considered in future research. Additionally, Indonesia's diverse sociodemographic makeup means these results may not be fully generalisable to the entire country.

## Conclusion

Our study found that our patients reported high body image scores, but diminished sexual functioning and enjoyment, per QLQ-BR-23. Early menopause and single status were associated with lower sexual QoL, while higher education and economic status improved sexual outcomes. Chemotherapy side effects were the most debilitating. MRM surgery was associated with higher body image, lower systemic therapy side effects and breast symptoms. Longer illness duration worsened arm symptoms, though unconventional treatments correlated with lower arm symptom scores.

However, the nature of the cross-sectional design of our study must always be considered when interpreting the results since causality cannot be established. These issues warranted the need for holistic, multidisciplinary care aimed at both medical and psychological support, especially on sexual issues for breast cancer patients, emphasising the need of a multidisciplinary approach that integrated oncological service, psychological services and supportive cares in addition to integrated economic support from both government and occupation-related social groups towards improving breast cancer patients’ QoL. Further studies can be performed to evaluate specified LABC care and usage of unconventional treatments in the future.

## List of abbreviations

HR-QoL, Health-related quality of life; LABC, locally advanced breast cancer; QLQ-BR23, The Quality-of-Life Questionnaire Breast Cancer 23; WHO, World Health Organisation; LMIC, Low-and middle-income countries; SPSS, Statistical Package for the Social Sciences; EORTC, European Organisation for Research and Treatment of Cancer; ANOVA, Analysis of Variance; MRM, Modified radical mastectomy; US, United States; NAC, Neoadjuvant chemotherapy.

## Ethics approval and consent to participate

The study obtained approval from the institutional ethics of the joint ethics committee from the Faculty of Medicine of Universitas Lambung Mangkurat/Ulin Regional Public Hospital with registry number 048/KEPK-FKIK ULM/EC/IV/2024. Written consent with the addition of a written signature from each of the participants had been taken prior to the interviews.

All methods were carried out in accordance with relevant guidelines and regulations. Both digital and written documents were collected and stored by the corresponding author and not shared to any third party outside our study group.

## Conflict of interest

The authors declare no competing interests.

## Author contributions

SHP: conceptualisation, methodology, critical reviewing, overall supervision; WB: methodology, critical reviewing, overall supervision, literature review, technical guidance; BT: methodology, critical reviewing, overall supervision; E: editing, data management, quality control of counselling, data quality assurance; MDIP: writing, data collection, data management.

## Figures and Tables

**Table 1.** Sociodemographic characteristics of LABC patients (*N* = 100).

Variables	*N*	%
Age		
Mean (±SD)	50 (±9.98)	
Duration of formal education (years)		
0–6 years	41	41
7–9 years	21	21
10–12 years	14	14
>12 years	24	24
Occupation		
Working	26	26
Not working	74	74
Marital status		
Married	79	79
Widow	19	19
Not Married	2	2
Monthly income		
Below provincial minimum wage	70	70
Above provincial minimum wage	30	30
Decline in savings		
Yes	67	67
No	33	33
Daily livelihood difficulties		
Yes	49	49
No	51	51

**Table 2.** Clinical characteristics of study participants (*N* =100).

Variables	*N*	%
IIIA	6	6
IIIB	89	89
IIIC	5	5
Duration of illness		
<1 year	39	43.2
1-5 years	51	44.4
>5 years	10	12.3
Hormonal treatment		
Yes	6	6
No	94	94
Chemotherapy treatment		
Yes	96	96
No	4	4
Surgical procedure (Modified-radical mastectomy)		
Yes	44	44
No	56	56
Unconventional medicines usage		
Yes	21	21
No	79	79

**Table 3.** Assessment of quality of life in locally advanced breast cancer patient by using QLQ-BR-23 questionnaire (*N* = 100).

Scales	*N*	No. of questionnaire items	Mean ± SD	95% CI	*N*(%) Scoring ≤33	*N*(%) Scoring ≥66	Median	Interquartile range	*p*-value
Functional scales (^*^									
Body image	100	4	74.58 ± 29.60	68.71–80.46	11	72	83.33	41.67	<0.001
Sexual functioning	100	2	31.00 ± 31.34	24.78–37.22	19	62	33.33	66.67	<0.001
Sexual enjoyment	100	2	24.99 ± 28.87	19.27–30.73	54	14	0.00	45.83	<0.001
Future perspective	100	1	48.33 ± 39.18	40.56–56.11	54	25	33.33	100	<0.001
Symptom scales (^**^									
Systemic therapy side effects	100	7	39.71 ± 20.78	35.59–43.83	35	16	40.48	32.14	0.019
Breast symptoms	100	4	22.58 ± 26.70	17.28–27.88	39	33	16.67	33.33	<0.001
Arm symptoms	100	3	19.44 ± 23.48	14.78–24.11	74	8	11.11	30.55	<0.001
Upset by hair loss	100	1	39.67 ± 38.69	31.99–47.34	69	11	33.33	66.67	<0.001

(* For functional scales, higher scores designate better functioning

(** For symptom scales, higher scores designate worse functioning

**Table 4.** Comparison of quality-of-life scores by using QLQ-BR-23 questionnaire between variable groups (*N* = 100).

Variables	Functional scales in QLQ-BR-23 (^*^				Symptoms scales in QLQ-BR-23 (^**^			
	Body image	Sexual functioning	Sexual enjoyment	Future perspective	Systemic therapy side effects	Breast symptoms	Arm symptoms	Upset by hair loss
Clinical staging								
IIIA (*N* = 6)	63.58	36.92	42.58	55.92	34.50	43.00	51.92	53.50
IIIB (*N* = 89)	49.42	51.28	51.29	50.22	51.87	50.69	50.02	51.10
IIIC (*N* = 5)	54.00	53.00	45.90	48.90	45.30	56.20	57.30	36.30
*p*-value	0.468	0.463	0.685	0.883	0.333	0.728	0.845	0.491
Age								
<50 years (*N* = 52)	53.70	52.88	54.23	53.07	48.09	47.66	45.63	50.17
≥50 years (*N* = 48)	47.03	47.92	46.46	47.72	53.11	53.57	55.78	50.85
*p*-value	0.234	0.372	0.145	0.341	0.385	0.294	0.069	0.902
Menopausal status								
Yes (*N* = 36)	47.97	58.01	52.56	49.4	55.9	53.5	54.88	56.67
No (*N* = 64)	51.92	46.27	49.34	51.12	47.46	48.81	48.04	47.03
*p*-value	0.499	**0.043**	0.563	0.769	0.161	0.424	0.240	0.095
Marital status								
Married (*N* = 79)	52.26	53.29	56.54	53.09	49.97	46.53	49.22	48.92
Widow (*N* = 19)	44.55	42.11	27.97	39.55	55.79	64.37	52.66	58.63
Not married (*N* = 2)	37.50	20.00	26.00	52.00	21.00	75.50	80.50	35.50
*p*-value	0.450	0.085	**0.000**	0.167	0.254	**0.021**	0.275	0.290
Duration of formal education								
0–6 years (*N* = 41)	48.71	47.85	41.48	50.13	50.12	49.87	49.93	52.60
7–9 years (*N* = 21)	49.62	51.07	53.26	46.55	53.00	56.60	58.26	44.19
10–12 years (*N* = 14)	58.82	53.61	59.68	58.86	54.82	46.64	34.96	53.46
>12 years (*N* = 24)	49.48	52.71	58.15	49.71	46.44	48.50	53.75	50.71
*p*-value	0.693	0.872	**0.036**	0.640	0.813	0.708	0.098	0.686
Occupational status								
Working (*N* = 26)	52.10	41.02	45.63	49.00	51.62	50.88	56.52	52.90
Not working (*N* = 74)	49.94	53.83	52.21	51.03	50.11	50.36	48.39	49.66
*p*-value	0.736	0.043	0.279	0.751	0.819	0.935	0.202	0.607
Economic status								
Below provincial minimum wage (*N* = 70)	48.26	47.29	45.90	49.92	52.38	55.16	53.44	51.55
Above provincial minimum wage (*N* = 30)	55.73	58.00	61.23	51.85	46.12	39.62	43.65	48.05
*p*-value	0.221	0.078	**0.008**	0.753	0.321	**0.011**	0.108	0.563
Duration of illness								
<1 year (*N* = 39)	44.77	44.86	48.29	48.31	57.14	53.21	58.15	56.51
1-5 years (*N* = 51)	54.53	54.36	52.10	51.49	45.94	50.22	42.95	46.73
>5 years (*N* = 10)	52.30	52.80	50.95	54.00	47.85	41.40	59.15	46.30
*p*-value	0.256	0.265	0.797	0.795	0.182	0.493	**0.022**	0.222
Has undergone surgery (Modified-radical mastectomy)								
Yes (*N* = 44)	58.63	53.39	53.40	54.86	43.07	43.82	48.93	45.60
No (*N* = 56)	44.12	48.23	48.22	47.07	56.34	55.75	51.73	54.35
*p*-value	**0.010**	0.358	0.335	0.168	**0.023**	**0.035**	0.619	0.117
Unconventional medicines usage								
Yes (*N* = 21)	48.93	45.50	46.19	52.52	43.19	48.38	38.45	42.62
No (*N* = 79)	50.92	51.83	51.65	49.96	52.44	51.06	53.70	52.59
*p*-value	0.772	0.354	0.405	0.710	0.193	0.698	**0.026**	0.143

(*) For functional scales, higher scores designate better functioning

(**) For symptom scales, higher scores designate worse functioning Bold *p*-values indicate statistically significant results; *p* < 0.05 (significant), *p* < 0.01 (highly significant), *p* < 0.001 (very highly significant)

**Table 5.** Effect sizes analysis of key statistical findings of table 6 depicting linear regression model with parameter for QLQ-BR-23 score schemes between variable groups (*N* = 100).

Outcome domain	R^2^	f^2^	Interpretation
Body image	0.148	0.174	Medium effect
Sexual functioning	0.232	0.302	Medium effect
Sexual enjoyment	0.3	0.429	Large effect
Future perspective	0.07	0.075	Small effect

**Table 6.** Linear regression model with parameter for QLQ-BR-23 score schemes between variable groups (*N* = 100).

	Body image				Sexual functioning				Sexual enjoyment				Future perspective			
	**Standardized Coefficient Beta**	**Significance**	**95% CI**		**Standardized Coefficient Beta**	**Significance**	**95% CI**		**Standardized Coefficient Beta**	**Significance**	**95% CI**		**Standardized Coefficient Beta**	**Significance**	**95% CI**	
			**Lower**	**Upper**			**Lower**	**Upper**			**Lower**	**Upper**			**Lower**	**Upper**
Constant	74.411	0.000	42.095	106.728	2.455	0.881	−30.036	34.947	−16.440	0.256	−45.011	12.131	35.145	0.122	−9.540	79.831
Clinical staging (Ref: IIIA)																
IIIB	−0.110	0.443	−36.937	16.310	0.159	0.244	−10.970	42.565	0.164	0.206	−8.450	38.626	0.003	0.986	−36.476	37.150
IIIC	−0.022	0.877	−41.066	35.121	0.013	0.926	−36.495	40.104	−0.013	0.920	−35.381	31.975	−0.027	0.858	−57.433	47.913
Age (Ref: <50 years old)																
≥50 years old	−0.022	0.527	−16.879	8.704	−0.091	0.381	−18.560	7.161	−0.031	0.758	−13.069	9.548	−0.071	0.536	−23.209	12.165
Menopausal status (Ref: no)																
Yes	−0.052	0.641	−16.703	10.333	0.233	0.029	1.576	28.759	0.063	0.531	−8.171	15.731	−0.019	0.870	−20.233	17.151
Marital status (Ref: not in marriage status)																
Married	−0.027	0.813	−18.087	14.236	0.054	0.616	−12.140	20.358	0.371	0.000	11.910	40.486	0.140	0.235	−8.904	35.790
Educational status (Ref: 0–6 years)																
7–9 years	−0.042	0.726	−20.254	14.164	−0.044	0.702	−20.637	13.967	0.156	0.154	−4.194	26.234	−0.070	0.578	−30.478	17.112
10–12 years	0.165	0.186	−6.875	34.835	−0.064	0.588	−26.705	15.232	0.156	0.167	−5.514	31.363	0.097	0.454	−17.921	39.754
> 12 years	−0.048	0.760	−24.834	18.194	−0.051	0.732	−25.368	17.892	0.216	0.133	−4.503	33.538	0.002	0.989	−29.548	29.948
Occupational status (Ref: not working)																
Working	0.084	0.483	−10.347	21.690	−0.246	0.033	−33.624	−1.415	−0.187	0.089	−26.429	1.894	0.003	0.979	−21.861	22.437
Economic status (Ref: Below minimum wage)																
Above minimum wage	0.124	0.365	−9.374	25.250	0.292	0.026	2.492	37.303	0.063	0.607	−11.333	19.278	−0.045	0.751	−27.764	20.113
Surgical procedure (Ref: no)																
Yes	0.209	0.056	−0.304	25.043	0.048	0.638	−9.716	15.769	0.064	0.514	−7.509	14.900	0.133	0.238	−7.048	28.000
Unconventional medicines usage (Ref: no)																
Yes	−0.091	0.397	−21.911	8.779	−0.155	0.131	−27.262	3.594	−0.143	0.143	−23.657	3.477	0.022	0.841	−19.073	23.363
*p*-value	0.412				**0.047**				**0.004**				0.949			
R^2^	0.148				0.232				0.300				0.070			
Adjusted R^2^	0.007				**0.105**				**0.185**				0.083			
Effect sizes interpretation	Fairly large effect				Fairly large effect				Fairly large effect				Small-medium effect			

Values are expressed as mean. Bold *p*-values indicate statistically significant results; *p* < 0.05 (significant), *p* < 0.01 (highly significant), *p* < 0.001 (very highly signiificant) Values expressed in Adjusted *R*^2^ indicate variance in QLQ-BR-23 scores obtained through linear regression
